# Prevalence of anxiety and depressive symptoms in ulcerative colitis patients in Jordan and its relationship to patient-reported disease activity

**DOI:** 10.1038/s41598-022-11911-4

**Published:** 2022-05-10

**Authors:** Awni Abu Sneineh, Sara Haj Ali, Ahmad Al-Masri, Amr Diab, Farah Aldweik, Mohammad Darweesh, Mohammad Qaisi, Osama Alshakhatreh, Tarek Tamimi, Yaser Rayyan, Radwan Banimustafa, Ibrahim Sablaban

**Affiliations:** 1 Department of Gastroenterology, Faculty of Medicine, University of Jordan, Amman, 11942 Jordan; 2 Department of Internal Medicine, Faculty of Medicine, Al-Balqa Applied University, Salt, 19117 Jordan; 3 Department of Internal Medicine, Faculty of Medicine, University of Jordan, Amman, 11942 Jordan; 4 Department of Psychiatry, Faculty of Medicine, University of Jordan, Amman, 11942 Jordan; 5 Department of Psychiatry, Henry Ford Hospital, Detroit, MI 48202 USA

**Keywords:** Gastroenterology, Gastrointestinal diseases, Psychiatric disorders

## Abstract

Inflammatory bowel disease is associated with higher rates of anxiety and depression compared to the general population. We aimed to determine the prevalence of anxiety and depressive symptoms among patients with ulcerative colitis and correlation to disease activity. In this cross-sectional study, we collected data from 70 consecutive ulcerative colitis patients over one year at our inflammatory bowel disease outpatient clinic through an interview and a questionnaire containing patient demographics and disease characteristics. Anxiety and depressive symptoms were characterized using the Generalized Anxiety Disorder-7 questionnaire and Patient Health Questionnaire-9, respectively, with ulcerative colitis disease severity assessed by the Partial Mayo scoring system. The majority of our patients were females (68.6%) and the mean age was 39.3 years. Rates of anxiety and depressive symptoms among ulcerative colitis patients were 65.7% and 58.6%, respectively. Depressive symptoms were significantly associated with patient-reported disease activity (*r* = 0.361; *p* = 0.010). Significant percentages of ulcerative colitis patients were appreciated to have anxiety and depressive symptoms, and there was a correlation between patient-reported disease activity and depressive symptoms. At this high rate of prevalence, it is justified to screen patients for the presence of psychiatric comorbidities.

## Introduction

Ulcerative colitis (UC) is a chronic inflammatory disease (a subtype of inflammatory bowel disease, IBD) affecting the lining of gastrointestinal tract that can lead to substantial debility. Typically afflicting those under the age of 30, the incidence and prevalence of UC ranges between 6.3–24.3/100,000 and 249–505/100,000, respectively, greatly clustering in Europe and North America. Incidence has notably been increasing over time^1^. The signs and symptoms vary and may include abdominal pain, diarrhea, rectal bleeding, fatigue, fever, and weight loss. Ultimately, UC accounts for a great decline in quality of life over the disease course^2,3^.

IBD has been demonstrated in the literature to pose great psychological burden to both patients and caretakers. This may be attributed to the chronicity of the disease, the distressing nature of symptoms, as well as debility in the generally younger population we see IBD in. This has been demonstrated in active as well as quiescent disease^2,4,5^.

The presence of psychiatric illness has been linked to adverse IBD outcome and higher health care cost^6,7^.

A previous systematic review by Antonina Mikocka-Walus et al. reported a prevalence rate of depression in IBD ranging between 7 and 59% while that for anxiety was between 15.1 and 40%^8^. A more recent systematic review and a meta-analysis by Barberio, Zamani et al. found that the prevalence of depressive symptoms in IBD was 25.2% while that of anxiety symptoms was 32.1%, the rates were higher in patients with active IBD and the risk was higher in patients with CD compared with UC^9^. Data from the Middle East are very limited on this topic with only few studies reporting rates of depressive and anxiety symptoms in IBD patients. A study from Israel on IBD patients reported depression rate of 9.36% and anxiety rate of 23.4%^10^. Another study from Iran found that anxiety, depression and psychological stress were highly frequent in UC patients with rates of 29.2%, 40%, and 38.3%, respectively^11^.

Alexakis et al. performed a systematic review of 11 studies assessing the level of depressive symptoms and disease course in IBD and found that 5 studies reported an association only in Crohn’s disease. This association was not apparent in patients whose IBD was in remission at baseline^12^.

A recent Canadian longitudinal cohort study by Marrie et al. found that within individuals, elevations in symptoms of depression over time were associated with increased odds of active IBD, but elevated symptoms of anxiety were not^13^.

In our study, we assessed anxiety and depressive symptoms in patients with UC in Jordan. No similar studies have been conducted in Jordan with very limited data from the Middle East.

## Materials and methods

We conducted a cross-sectional study in which we contacted 70 consecutive patients with UC in gastroenterology department of Jordan University Hospital. Patients who were diagnosed with UC by a gastroenterologist clinically, endoscopically and confirmed with histopathology were recruited to the study from October 2017 to October 2018.

The study took place in the outpatient clinics of the gastroenterology and hepatology department at Jordan University Hospital in Amman. Each participant was interviewed by one of the researchers and asked questions to assess disease activity and measure anxiety and depressive symptoms level.

The questionnaire consisted of demographical data (age, gender, education, employment, etc.) and incorporated The Partial Mayo Scoring Index (PMSI)^14^, and other questions (duration of IBD disease, number of flares, related surgeries, type of treatment, whether the patients took antidepressants, or have visited a psychiatrist). The Patient Health Questionnaire-9 (PHQ-9) was used to assess depressive symptoms score with a sensitivity and specificity of 88% for major depression in those scoring 10 points^15^. The Generalized Anxiety Disorder-7 (GAD-7) score was used to assess for severity of anxiety symptoms, developed by Spitzer, Williams and Kroenke and validated in several studies with a sensitivity of 89% and specificity of 82%^16^.

### Measurements

#### Anxiety and depression

Anxiety symptoms level was measured using the GAD-7. The GAD-7 contains 7 questions graded on a 4-point Likert scale (0–3), with a sum score ranging from 0 to 21^16^. Patients who scored more than 4 were considered to have anxiety symptoms. A score of 5–9 for mild, 10–14 for moderate, and > 15 for severe anxiety symptoms. Depressive symptoms were characterized using the PHQ-9, which contains 9 questions graded on a 4-point Likert scale (0–3), with a sum score ranging from 0 to 27. Patients who scored more than 4 were considered to have depressive symptoms.

A score of 5–9 for mild, 10–14 for moderate, 15–19 for moderately severe and > 20 for severe depressive symptoms^15^.

#### Disease activity

Patient-reported disease activity in the participants was assessed by the PMSI. PMSI subscores of 0 were considered as normal, subscores of 1 were considered as mild disease, subscores of 1 to 2 were considered as moderate disease and subscores of 2 to 3 were considered as severe disease^14,17^.

### Statistical analysis

Statistical analyses were performed using Statistical Package for Social Science (IBM SPSS Statistics; Version 21.0, version for Windows, 2019, USA). Frequencies and percentages were used to describe categorical variables using frequencies method. Mean and standard deviation (SD) were calculated for the continuous variables. The frequency of participants in terms of categorical variables was compared using X^2^ test. Spearman’s rho test was calculated to assess the relationship between depressive symptoms, anxiety symptoms and patient-reported disease activity measured categorially. A *p* value < 0.05 was considered as statistically significant.

### Ethical considerations

The study protocol was approved by the Ethics Committee at Jordan University hospital and was conducted in accordance with the 1964 Helsinki Declaration. Informed consent was obtained from all individual participants involved in the study.

## Results

### Patients characteristics

The majority of the participants were females (68.6%) and 68.6% of all patients were married. The mean age of participants was 39.3 years (range 16–68 years) and (47.1%) were unemployed. Five patients (7.1%) had a colectomy. The mean number of years since diagnosis was 10.6 years. At the time of interview, nearly half of the patients were in remission (51.4%) and only four (5.7%) had severe disease (Table 1).

**Table 1:** Patient and disease characteristics of 70 consecutive patients with ulcerative colitis.

Female sex, n (%)	48 (68.6)
Age, years (mean, SD)	39.3 ± 13.64
**Education, n (%)**	
High school degree	23 (32.9)
College education	6 (8.6)
Bachelor’s degree	29 (41.4)
Graduate degree	12 (17.1)
**Employment, n (%)**	
Unemployed	33 (47.1)
Employed	26 (37.1)
Retired	11 (15.7)
**Marital status, n (%)**	
Single	22 (31.4)
Married	48 (68.6)
Disease duration, years (mean, SD)	10.6 ± 8.89
**Medications**	
Steroids, n (%)	6 (8.6)
Current/previous biologics, n (%)	14 (20)
Active disease, n (%)	34 (48.6)
Colectomy n (%)	5 (7.1)

SD, standard deviation.

Overall, the mean GAD-7 score was 8.63 (range 0–21) and the mean PHQ-9 score was 6.87 (range 0–22). We found that forty-six (65.7%) participants scored above the normal threshold for anxiety symptoms. Fifteen patients (21.4%) had mild, eighteen (25.7%) had moderate and thirteen (18.6%) had severe anxiety symptoms.

Forty-one (58.6%) of the sample had depressive symptoms, divided as follows: twenty-one (30%) had mild, sixteen (22.9%) had moderate, three (4.3%) had moderately severe and one patient (1.4%) had severe depressive symptoms (Table 2).

**Table 2:** Anxiety and depressive symptoms scores in 70 consecutive patients with UC.

GAD-7 SCORE (Mean ± SD)	8.63 ± 5.89	PHQ-9 SCORE (Mean ± SD)	6.87 ± 4.975
	N (%)		N (%)
Below the normal threshold for anxiety (less than 5)	24 (34.3)	Below the cut-off point to have depression (0–4)	29 (41.4)
Mild anxiety symptoms (5–9)	15 (21.4)	Mild depressive symptoms (5–9)	21 (30)
Moderate anxiety symptoms (10–14)	18 (25.7)	Moderate depressive symptoms (10–14)	16 (22.9)
Severe Anxiety symptoms (15 and above)	13 (18.6)	Moderately severe depressive symptoms (15–19)	3 (4.3)
		Severe depressive symptoms (20–27)	1 (1.4)

*UC* ulcerative colitis, *GAD-7* generalized anxiety disorder-7, *PHQ-9* patient health questionnaire-9, *SD* standard deviation.

We assessed the relationship between depressive symptoms (measured as PHQ-9 score), anxiety symptoms (measured as GAD-7 score) and patient-reported disease activity measured categorically using Spearman’s rho test. The association between patient-reported disease activity and depressive symptoms was found to be significantly moderate (*r* = 0.361; *p* = 0.010; Table 3), while the correlation between patient-reported disease activity and anxiety symptoms showed a significantly weak association (*r* = 0.252 and *p* = 0.035). Also, there was a significant association between depressive and anxiety symptoms (*r* = 0.636; *p* = 0.001). We performed the analysis with and without patients who had surgery and obtained the same results.

**Table 3:** Correlations between patient-reported disease activity, anxiety and depressive symptoms as categorical variables among UC Patients (Spearman’s rho test).

	Patient-reported disease activity (UC)	GAD-7 score	PHQ-9 score
**Patient-reported disease activity**			
*r*		0.252^†^	0.361^‡^
*p* value		0.035	0.010
**GAD-7 score**			
*r*	0.252^†^		0.636^‡^
*p* value	0.035		0.001
**PHQ-9 score**			
*r*	0.361^†^	0.636^‡^	
*p* value	0.002	0.001	

*UC* ulcerative colitis, *GAD-7* generalized anxiety disorder-7, *PHQ-9* patient health questionnaire-9.

^†^Correlation is significant at the 0.05 level (2-tailed).

^‡^Correlation is significant at the 0.01 level (2-tailed).

## Discussion

Our data is largely consistent with the results of other studies on this topic, which have found anxiety and depressive symptoms to be more common in patients with IBD than in the general population.

Our study demonstrated that the prevalence of anxiety and depressive symptoms in UC patients was 65.7% and 58.6%, respectively, which is higher than that reported in the general Jordanian population. There are only two local studies that examined the prevalence of anxiety and depression among Jordanians and reported rates of 23.7% and 13.3%, respectively^18,19^. Remarkably, our study demonstrated higher rates of depressive and anxiety symptoms in our population compared to what we found in the literature reported from other countries. According to a recent meta-analysis, the prevalence of depression in IBD patients was 25.2% while that for anxiety was 32.1%^9^. Similar to our study, Fakheri et al. also reported high rates of anxiety and depression in UC patients in Iran (81.5% and 43.5%, respectively)^20^. We attribute this difference as likely caused by varied perceptions and comfort with mental illness in populations studied, differences in methods used to assess depression and anxiety and different cut-off points used to diagnose anxiety and depressive symptoms using the same questionnaires. The use of screening questionnaires may overestimate rates of depression and anxiety. In addition, some of the depressive symptoms used in the questionnaires like fatigue and change in appetite may overlap with IBD symptoms, therefore, the PHQ-9 may overestimate depression in IBD patients. Also, the majority of our patients were females, and female sex has been reported to be associated with increased risk of anxiety in studies of IBD patients^21^ as well as in the general population^22^.

A previous systematic review and meta-analysis by Alexakis et al. which looked at studies that assessed the impact of depressive symptoms on disease course in IBD found an association only in CD^12^. Byrne et al. studied the prevalence of anxiety and depression in IBD patients and found that disease activity was significantly associated with depression and/or anxiety^23^. Another study by Marrie et al. examined the association between elevated symptoms of depression and anxiety and disease activity in IBD and found that patients with elevated symptoms of depression and anxiety had increased odd of active IBD^13^. Similar to these results, our study found that patient-reported disease activity was associated with increased risk of depressive symptoms while the association with anxiety symptoms was weak. There was also a significant association between depressive and anxiety symptoms (Table 3).

The most probable explanation for this result is that depression and anxiety are reactions to the IBD, because the physical symptoms of the IBD itself including chronic diarrhea, abdominal pain, general weakness, and easy fatigability prevent the patients from carrying on their normal social and occupational life. Moreover, symptoms of irritable bowel syndrome (IBS) may overlap with symptoms of IBD which may contribute to the development of anxiety and depression specially in those in disease remission given IBS’ frequent comorbidity with clinical depression^24^. Medication side effects could also provide an explanation for our findings, with corticosteroids, commonly used for treatment of IBD, having strong associations with psychiatric illness and the exacerbation of depressive symptoms^25^. Finally, patients are burdened with the challenges of the impact of the disease itself, including effects on intimacy, other clinical complications and social stigmatization^26^.

The limitations of the study include the relatively small number of patients recruited and the fact that the patients were selected from a specialized clinic in a tertiary referral hospital which may have resulted in patients with more severe or active disease compared to community samples. Another limitation is that the psychological symptoms were assessed at the current patient’s status. We did not evaluate lifetime, 12-months risk or changes in psychological status over time. We used patient-reported disease activity and did not measure disease activity using a biomarker, therefore, it is uncertain whether active disease reflected active symptoms due to inflammation or simply active symptoms associated with IBS. However, two thirds of all the patients in our study were females, and unlike other studies, there was no selection bias as all consecutive patients in the clinic agreed to join the study and were recruited^27^. The interviews with each patient were conducted in person and we used the most up-to-date parameters to evaluate patient PHQ-9 and GAD-7 scores.

## Conclusion

Our study has shown that significant percentage of UC patients suffer from anxiety (65.7%) and depressive symptoms (58.6%). There was a correlation between patient-reported disease activity and depressive symptoms, the correlation with anxiety symptoms was less prominent. At this high rate of prevalence, it is justified to screen these patients for the presence of psychiatric comorbidities, especially those with active disease which is often unrecognized and untreated. This might improve the quality of life for patients with UC and promote their mental wellbeing.

## Data Availability

The datasets generated during and/or analysed during the current study are available from the corresponding author on reasonable request.
